# HDL cholesterol: reappraisal of its clinical relevance

**DOI:** 10.1007/s00392-017-1106-1

**Published:** 2017-03-24

**Authors:** Winfried März, Marcus E. Kleber, Hubert Scharnagl, Timotheus Speer, Stephen Zewinger, Andreas Ritsch, Klaus G. Parhofer, Arnold von Eckardstein, Ulf Landmesser, Ulrich Laufs

**Affiliations:** 1 Medizinische Klinik V (Nephrologie, Hypertensiologie, Rheumatologie, Endokrinologie, Diabetelogie), Medizinische Fakultät Mannheim der Universität Heidelberg, Heidelberg, Germany; 2 Klinisches Institut für Medizinische und Chemische Labordiagnostik, Medizinische Universität Graz, Graz, Austria; 3 Synlab Akademie, synlab Holding Deutschland GmbH, Mannheim und Augsburg, Augsburg, Germany; 4 Klinik für Innere Medizin IV, Nieren- und Hochdruckkrankheiten, Universitätsklinikum des Saarlandes, 66421 Homburg, Saarland Germany; 5 Klinik für Innere Medizin, Medizinische Universität Innsbruck, Innsbruck, Austria; 6 Medizinische Klinik II, Klinikum der Universität München, 81377 Munich, Germany; 7 Institut für Klinische Chemie, Universitäts Spital, 8091 Zurich, Switzerland; 8 Klinik für Kardiologie, Charité, Berlin, Germany; 9 Klinik für Innere Medizin III, Kardiologie, Angiologie und Internistische Intensivmedizin, IMED, Universitätsklinikum des Saarlandes, 66421 Homburg, Saarland Germany; 10 Institut für Ernährungswissenschaften, Friedrich Schiller Universität Jena, Jena, Germany

**Keywords:** HDL, Cholesterol, Review

## Abstract

**Background:**

While several lines of evidence prove that elevated concentrations of low-density lipoproteins (LDL) causally contribute to the development of atherosclerosis and its clinical consequences, high-density lipoproteins are still widely believed to exert atheroprotective effects. Hence, HDL cholesterol (HDL-C) is in general still considered as “good cholesterol”. Recent research, however, suggests that this might not always be the case and that a fundamental reassessment of the clinical significance of HDL-C is warranted.

**Method:**

This review article is based on a selective literature review.

**Results:**

In individuals without a history of cardiovascular events, low concentrations of HDL-C are inversely associated with the risk of future cardiovascular events. This relationship may, however, not apply to patients with metabolic disorders or manifest cardiovascular disease. The classical function of HDL is to mobilise cholesterol from extrahepatic tissues for delivery to the liver for excretion. These roles in cholesterol metabolism as well as many other biological functions of HDL particles are dependent on the number as well as protein and lipid composition of HDL particles. They are poorly reflected by the HDL-C concentration. HDL can even exert negative vascular effects, if its composition is pathologically altered. High serum HDL-C is therefore no longer regarded protective. In line with this, recent pharmacological approaches to raise HDL-C concentration have not been able to show reductions of cardiovascular outcomes.

**Conclusion:**

In contrast to LDL cholesterol (LDL-C), HDL-C correlates with cardiovascular risk only in healthy individuals. The calculation of the ratio of LDL-C to HDL-C is not useful for all patients. Low HDL-C should prompt examination of additional metabolic and inflammatory pathologies. An increase in HDL-C through lifestyle change (smoking cessation, physical exercise) has positive effects and is recommended. However, HDL-C is currently not a valid target for drug therapy.

## Epidemiology

**Fig. 1:**
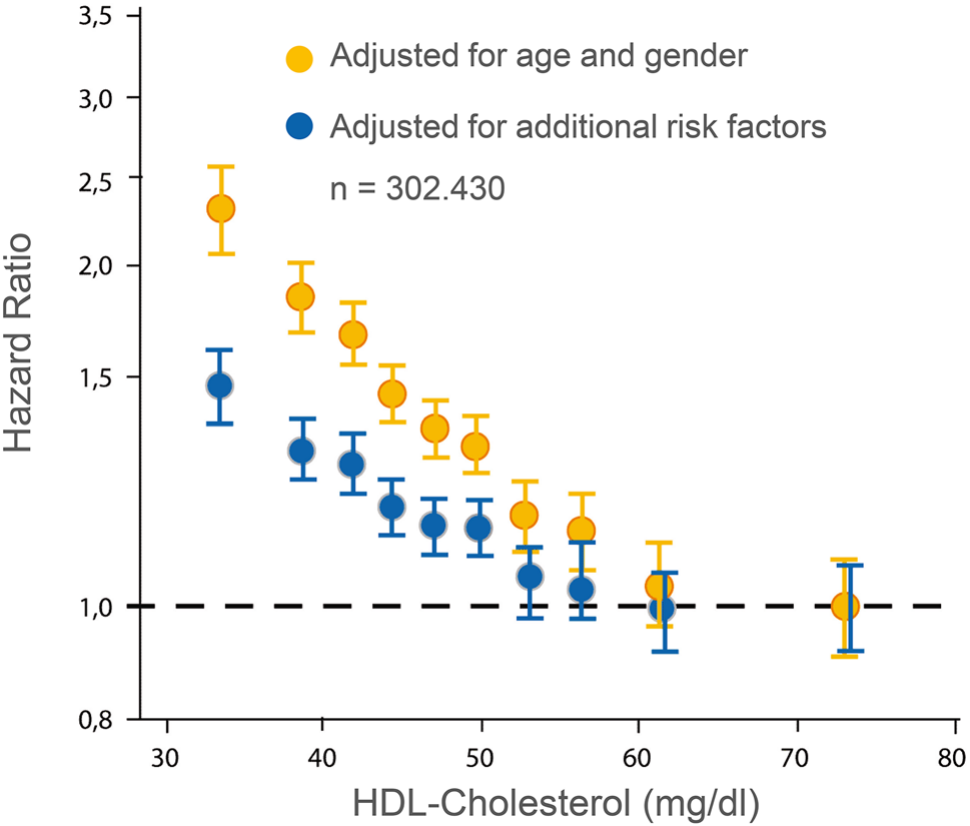
Epidemiological association of low HDL-C serum concentrations with CHD risk. Data from the Emerging Risk Factors Collaboration [3]. With permission of Springer [7]

The concept that high-density lipoproteins (HDL) could protect against coronary heart disease (CHD) primarily originated from epidemiological studies of the healthy population, in particular the Framingham study [1]. Patients with manifest CHD frequently exhibit low HDL cholesterol (HDL-C) [2]. The relationship between HDL-C and cardiovascular risk is not linear; for instance, no further improvement in prognosis is seen with HDL-C levels above ~60 mg/dl (1.5 mmol/l) (Fig. 1) [3]. Retrospective investigations of the EPIC Norfolk and of the IDEAL study show that very high concentrations of HDL-C may be associated with increased risk [4]. A recent register study of more than 1 million US veterans found a U-shaped relationship between HDL-C and total mortality with 50 mg/dL (1.25 mmol/L) as the nadir associated with the lowest mortality [5]. A very recent analysis of the Framingham study reports that the predictive value of HDL-C is modified by LDL cholesterol (LDL-C) and triglycerides (TG): Compared to low HDL-C (defined as <50 mg/dl in women and <40 mg/dl in men) in isolation, risk increases when low HDL-C occurs together with high LDL-C and/or TG. Cardiovascular (CV) risk increases by 30% for LDL-C ≥ 100 mg/dl and TG < 100 mg/dl or LDL-C < 100 mg/dl and TG ≥ 100 mg/dl. When both TG and LDL-C are ≥100 mg/dl, CV risk increases by 60% [6].

*In summary* Low HDL-C is an indicator of increased cardiovascular risk, especially in persons without a history of cardiovascular events; however, the epidemiological relationship between HDL-C and risk is complex. Reduced HDL-C concentrations are frequently confounded with other pro-atherogenic conditions, notably the presence of inflammation and pro-atherogenic triglyceride-rich lipoproteins and their remnants as well as small dense LDL. The continued widespread practice of calculating the ratio of LDL-C to HDL-C is not useful, because high HDL-C is not associated with reduced risk, so that a combination of high LDL-C and HDL-C may lead to the wrong conclusion that risk is not elevated.

## Role of HDL in lipoprotein metabolism

**Fig. 2:**
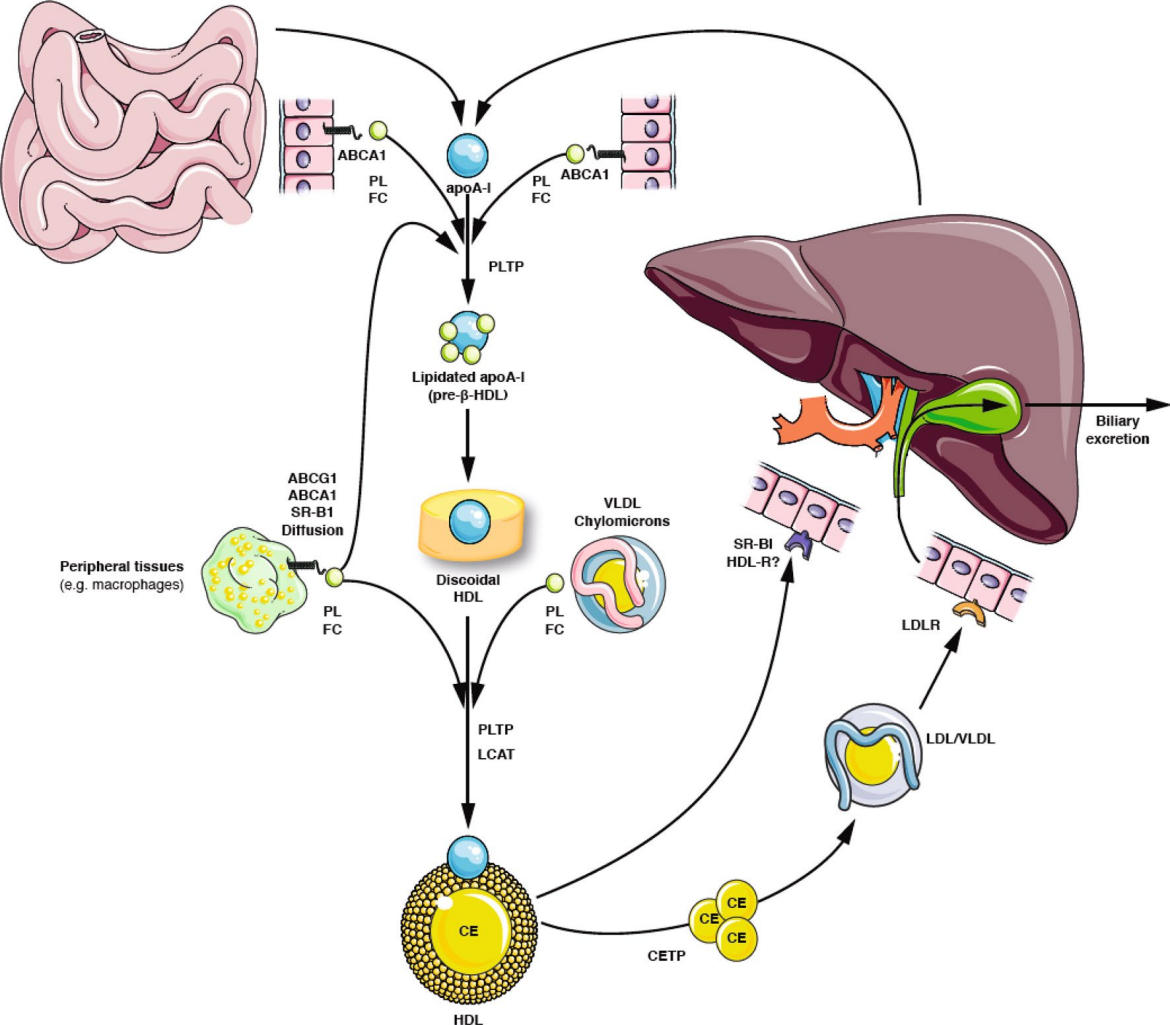
Role of HDL in lipoprotein metabolism. Apo A1, the main protein in HDL, is formed in the liver and the small intestine and secreted as lipid-free pre-ß-HDL. Pre-b-HDL can also come from chylomicron metabolism, or from the interconversion of HDL itself. Their interaction with ATP-binding cassette transporter A1 (ABCA1) leads to the efflux of phosphatidylcholine (PC) and free cholesterol (FC), and thus to the formation of disc-shaped HDL. Esterification of the free cholesterol by lecithin–cholesterol acyltransferase (LCAT) leads to the formation of mature spherical HDL. The lipid-rich discoidal and spherical HDL enable passive diffusion out of cells, which is facilitated by ATP-binding cassette transporter G1 (ABCG1), scavenger receptor class B type I (SR-B1) and by cholesterol esterification mediated by LCAT. Cholesteryl ester transfer protein (CETP) transfers cholesteryl esters (CE), triglycerides and phospholipids (PL) between HDL, LDL and VLDL. It thereby contributes to the formation of LDL, which is taken up through LDL receptors (LDLR) in hepatocytes. Phospholipid transfer protein (PLTP) belongs to the same protein family as CETP. Its function is to transport phospholipids between HDL and VLDL, and between various HDL. Mediated by SR-BI, HDL can deliver cholesteryl ester (and free cholesterol) to hepatocytes, steroid-producing cells and adipocytes. With permission of Springer [7]

HDL are the smallest (5–17 nm) and densest (1.063–1.210 kg/l) lipoproteins in the plasma. Apolipoprotein (Apo) A1, the major protein in HDL, is synthesised in the liver and the small intestine. The liver is the most important organ through which cholesterol is excreted, either directly or after being converted into bile acids. Excess cholesterol is transported from the periphery (e.g. from macrophages in blood vessel walls) to the liver. HDL play a key role in this pathway, known as reverse cholesterol transport (RCT) (Fig. 2) [8–11]. In addition to HDL, LDL also significantly contribute to RCT. The overwhelming majority of HDL-C measured in the blood originates from the liver and the intestine. Therefore, the concentration of HDL-C in the plasma cannot be used as a measure of cholesterol efflux from vessel walls, or of the efficiency of RCT.

The RCT begins with the transfer of cholesterol from cell membranes to HDL. To date, four biochemical pathways have been described that are involved in this transfer [8]. As long as the cholesterol content of the cell is normal, more than two-thirds of the (poorly water-soluble, non-esterified) cholesterol leave the cells by passive diffusion along a concentration gradient between the cell membrane and preferably large, globular HDL. This concentration gradient is maintained by extracellular esterification of free cholesterol mediated by the lecithin cholesterol acyltransferase (LCAT).

Secondly, the passive, aqueous efflux of non-esterified cholesterol can be further enhanced by up-regulation of ATP-binding cassette transporter G1 (ABCG1). ABCG1 mobilises cholesterol from subcellular compartments.

Thirdly, passive, but non-aqueous transfer of free cholesterol from cells to larger HDL can be mediated by the scavenger receptor class B type I (SR-B1). SR-B1 promotes not only the cellular cholesterol efflux, but also the “selective” (i.e. non-endocytotic) delivery of cholesterol from HDL to liver cells [12].

Fourthly, cholesterol and phospholipids from macrophages and foam cells are actively transferred to ATP-binding cassette transporter A1 (ABCA1) to lipid-free apo A1 (pre-ß-HDL). This process is important for the formation of “nascent” HDL particles, (i.e. HDL discs), which contain Apo A1, phosphatidylcholine and non-esterified cholesterol. The importance of ABCA1 for the metabolic maturation of HDL was identified by the elucidation of the genetic cause of Tangier’s disease [13, 14]. The expression of ABCA1 is regulated by the intracellular cholesterol content, and efflux mediated by ABCA1 is therefore important in situations of cellular cholesterol excess.

The directed flux of cholesterol between cells and HDL is dependent on a concentration gradient between the cell membrane and acceptor lipoproteins. This gradient is maintained at the cellular level by the hydrolysis of cholesteryl esters and translocation of cholesterol, extracellularly by lecithin cholesterol acyltransferase (LCAT) and the cholesterol ester transfer protein (CETP). The LCAT esterifies free cholesterol (for example, cholesterol associated with pre-ß-HDL) with a fatty acid from the lecithin. Cholesteryl esters are more hydrophobic than free cholesterol and are located inside the HDL. This transforms HDL from discoidal into spherical, pseudomicellular particles (α-HDL). Maturation of HDL is incomplete in genetic or secondary deficiency of LCAT. In patients with familial LCAT deficiency, spherical HDL are absent.

CETP is a hydrophobic glycoprotein which is mainly secreted by the liver and is mostly bound to HDL in the blood [15]. It facilitates the transfer of cholesteryl esters, triglycerides and, to a smaller extent, phospholipids between HDL, LDL and VLDL. Thus, cholesteryl esters are channelled into the metabolism of LDL and delivered to the liver.

*In summary* HDL-particles play a crucial role in the reverse transport and excretion of cholesterol. However, HDL-cholesterol measurements do not reflect HDL function in RCT.

## Determination of HDL cholesterol in the clinical laboratory

The standard method for the determination of cholesterol in LDL and HDL is the combined method of precipitation and ultracentrifugation (beta quantification) [16]. In the first step, VLDL is recovered by means of ultracentrifugation, then LDL is precipitated and finally HDL-C is determined enzymatically in the supernatant. This method is not suitable for use in the clinical laboratory because of high costs and complexity. In the late 1990s, “homogeneous” methods were introduced which allow the measurement of HDL-C in a single reaction vessel with no prior separation steps [17]. In these methods, the cholesterol in non-HDL particles is “masked” with antibodies, polymers or detergents, and in a second reaction step HDL-C is determined enzymatically. The commercially available homogeneous assays show a good agreement with the reference method in normolipidemic samples and in many cases of hypercholesterolemia. The determination of HDL-C is hardly affected by prior consumption of food and the results can usually well be interpreted in post-prandial samples [18]. Differences to the reference method and between the assays are, however, observed in hypertriglyceridemia (elevated concentrations of chylomicrons and/or VLDL), at low HDL-C (<20 mg/dl), and if atypical lipoproteins are present (type III hyperlipoproteinemia, liver disease, chronic kidney disease) [19–21]. In these cases, HDL-C can be accurately measured by lipoprotein electrophoresis, however by staining lipoproteins using enzymatic cholesterol oxidation rather than unspecific lipophilic chemicals.

Due to the limitations of conventional lipoprotein analysis, researchers are considering the replacement of HDL-C and LDL-C determinations with the determination of Apo A1 and ApoB, respectively. Measurement of apolipoproteins can be readily standardised [22] and is not significantly distorted by high triglycerides. Apo B not only reflects the concentration of LDL, but all atherogenic lipoproteins taken together [VLDL, remnants, LDL, Lp(a)].

In recent years, assays have been introduced which reflect the functionality of HDL better than HDL-C. Of importance are the determination of HDL particle concentrations (and sizes) with nuclear magnetic resonance (NMR) spectroscopy [23], the measurement of HDL proteins (Apo A1, serum amyloid A) and the in vitro measurement of the cholesterol uptake capacity of HDL in cell culture models (cholesterol efflux) [24–27]. While the latter will most likely remain a research method, NMR and apolipoproteins are in principle suitable for routine use, once their standardisation has been accomplished.

*In summary* Current clinically available methods are able to determine the cholesterol content of HDL particles, but not their biological function. Standardised determinations of HDL-C, and even less so of assays for cholesterol efflux capacity, HDL particle number determination by NMR spectroscopy and HDL proteins, are still not satisfactory.

## Lesions from genetic abnormality of HDL metabolism

Table 1 provides an overview of rare monogenic disorders in the HDL metabolism. According to the results from Danish and American population studies, approximately 10% of individuals with HDL-C levels below the 5th percentile are heterozygous for mutations in the genes of APOA1, ABCA1 or LCAT [28, 29]. Data on the risk of atherosclerosis in these individuals are contradictory [30]. Some mutations of APOA1 have been associated with increased risk of myocardial infarction [31], and another one, Apo A1-Milano, may reduce the risk. Some mutations of the APOA1 gene also cause familial amyloidosis. In large Danish population studies, heterozygosity for HDL-C-lowering mutations of the ABCA1 or APOA1 gene was not associated with an increased risk of myocardial infarction. Interestingly, ABCA1 mutations increase the risk for Alzheimer’s disease. Data from large Dutch family studies suggest an increased cardiovascular risk of HDL-C-lowering mutations in the genes of APOA1, ABCA1 or LCAT. Italian studies found no evidence for an increased risk of atherosclerosis in heterozygous carriers of LCAT mutations [32–34].

**Table 1:** Monogenic forms of HDL deficiency

Mutatedgene	Clinical signs with 2 mutated alleles (homozygosity, comp. heterozygosity)	Clinical signs with 1 mutated allele (heterozygosity)
APOA1	ApoA-I deficiency *(OMIM 107680)* Plane xanthoma Corneal opacity Early-onset atherosclerosis	Familial amyloidosis in some variants *(OMIM 105200)* (early CHD possible)
ABCA1	Tangier disease *(OMIM 205400)* Orange tonsils Peripheral neuropathy Hepatosplenomegaly Early-onset atherosclerosis	None (Early CHD possible)
LCAT	LCAT deficiency *(OMIM 245900)* Corneal opacity Nephropathy (proteinuria, GFR↓)	None (Early CHD possible)
	Fish-eye disease *(OMIM126120)* Corneal opacity	

Homozygous and heterozygous carriers of mutations in these three genes are very rare and present themselves with pronounced or even complete HDL deficiency and characteristic clinical syndromes: patients with complete LCAT deficiency develop corneal opacities, anaemia and progressive renal failure, which ultimately requires renal replacement therapy. Patients with partial LCAT deficiency (“fish eye disease”) develop corneal opacities, but neither renal disease nor anaemia. In both cases, the remaining HDL are discoidal in shape. In classical LCAT deficiency, LDL include particles which are similar to Lipoprotein X and may be the cause of the characteristic nephropathy. The risk of atherosclerosis of mutation carriers is not increased or at most slightly increased [35], possibly because HDL can also transport free cholesterol directly to the liver [36].

Homozygosity for mutations in the ABCA1 gene leads to Tangier disease [13, 14]. As a result of the strongly reduced cellular cholesterol efflux, cholesterol accumulates in macrophages in various organs (enlarged tonsils, hepatosplenomegaly and peripheral neuropathy). Patients with Tangier disease often also have low LDL-C, which could attenuate the atherogenic effect of the low HDL concentration [Schaefer, 2010 # 21]. Homozygous nonsense mutations in the APOA1 gene were found to [28] be the cause of HDL deficiency in patients with severe xanthomatosis and early atherosclerosis.

Most patients with a deficiency of cholesteryl ester transfer protein (CETP) have been observed in Japan. Affected persons have very high concentrations of HDL-C. Pharmacological inhibition of CETP has therefore been tested as an approach to increase HDL. It is unclear whether the HDL produced through CETP inhibition have a normal function. We observed a slightly increased cardiovascular mortality at low concentrations of CETP in the blood despite high HDL-C [37].

Only recently, mutations of scavenger receptor (SR-BI) (P279S, P376L) have been described which are associated with both significant increases in HDL-C and increased cardiovascular risk [38–40]. These mutations impair the selective uptake of cholesteryl esters by hepatocytes. In mice, the overexpression of SR-B1 leads to an increase in RCT, despite a drop in HDL-C [41]. On the other hand, lack of SR-BI increases HDL-C, but is atherogenic [42]. High HDL-C therefore does not provide protection from atherosclerosis in all cases.

This stands in contrast to genetic factors which increase LDL cholesterol, because monogenetic (familial hypercholesterolemia) and polygenetic factors increasing LDL cholesterol consistently lead to concordant changes in the CV risk (positive Mendelian randomisation) [43, 44].

*In summary* Current genetic data do not support the concept of a general protective role for HDL-C with regard to coronary heart disease.

## Disorders with reduced HDL concentration

**Fig. 3:**
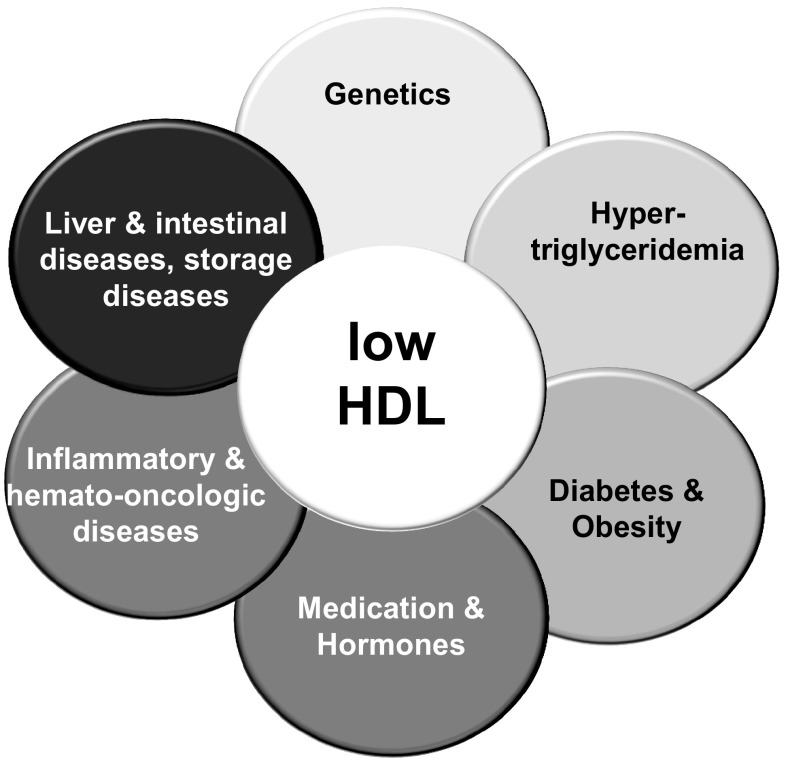
Aetiologies of low HDL cholesterol. With permission of Springer [7]

Compared to genetically determined abnormalities in HDL metabolism, low HDL-C much more frequently occur in patients with metabolic syndrome or diabetes mellitus. Low HDL-C levels are also associated with systemic inflammation, e.g. with cigarette smoking, chronic inflammatory diseases or chronic kidney disease (Fig. 3) [45, 46]. In cases of extremely low HDL-C, rare diagnosis may be considered, e.g. neoplasia or an increased risk for sepsis [47, 48].

*In summary* Low HDL-C is an indicator that the affected individual should be examined for metabolic and inflammatory pathology.

## Vascular protective function of HDL

Evidence has long been available from animal experiments for anti-atherosclerotic effects of HDL. Administration of homologous HDL in addition to an atherogenic diet inhibits the formation of fatty streaks in rabbits [49]. Infusion of Apo A1 Milano (R173C, complexed with phospholipids) inhibits atherogenesis in cholesterol-fed rabbits [50] and apo E-deficient mice [51].

Alongside its prominent role in RCT, anti-oxidative and anti-inflammatory effects are also attributed to HDL, as well as improvement of endothelial function [9, 52, 53]. They inhibit the pathological attachment of monocytes to the endothelium and support endothelial repair mechanisms. Many of the endothelial effects of HDL are mediated by the endothelial SR-B1 receptor and by the bioactive lipid sphingosine-1-phosphate that can act via the S1P3 receptor on endothelial cells [54]. Investigations of the composition of HDL [55, 56] have found enzymes, acute-phase proteins, components of the complement system and protease inhibitors in addition to the characteristic apolipoproteins associated with HDL. The lipidome of HDL is even more heterogeneous, and HDL can also function as the vehicle for microRNA in the blood [57]. Paraoxonase 1 (PON1) is an esterase associated with HDL, which inhibits the formation of lipid peroxides in LDL [58, 59] and HDL. Compared to healthy people, the activity of HDL-associated PON1 is reduced in patients with CHD [60]. An anti-oxidative effect is also attributed to Apo A1 itself [61]. HDL are anti-inflammatory in several ways: they inhibit the expression of adhesion molecules (VCAM-I and ICAM-I) in the endothelium, inhibit the terminal complement complex, inhibit the synthesis of chemokines (MCP-1) and induce the expression of the anti-inflammatory cytokine transforming growth factor beta 2.

In addition, HDL can have antithrombotic and profibrinolytic effects [62]. HDL protect from cell damage, necrosis and apoptosis. These protective effects have also been demonstrated in pancreatic beta cells, implying that HDL might be able to improve insulin secretion and protect from the development of diabetes mellitus [63–68].

*In summary* The anti-inflammatory, cytoprotective and wound-healing effects of HDL make them a part of the innate host defense system [69]. From an evolutionary point of view, the potential anti-atherogenic effects, if any of them exist, would likely be secondary.

## HDL dysfunction

Interestingly, several studies have provided evidence that the vascular effects of HDL are variable and hardly correlate with HDL-C concentrations in the plasma. In patients with diabetes mellitus, coronary disease, chronic renal insufficiency, cardiovascular risk factors and disorders, the function of HDL is impaired [9, 61, 70–74]. In patients who underwent coronary angiography, we have observed that HDL-C correlates inversely with cardiovascular mortality in the absence of CHD, that the relationship of HDL-C to long-term prognosis was weakened in stable coronary heart disease, whereas in clinically unstable patients the relationship was completely abolished [74].

The protein composition of HDL changes significantly in the course of an acute-phase reaction. The “acute-phase HDL” are characterised, for example, by increased concentrations of serum amyloid A (SAA), secretory phospholipase A2 (sPLA2-IIa) and ceruloplasmin, and a lower proportion of Apo A1 [73]. Unlike HDL from healthy subjects, HDL from patients with CHD, kidney disease or diabetes mellitus have no protective vascular effects or may even cause paradoxical harmful effects [55, 56, 61, 70, 71, 75, 76]. The characterisation of the HDL’s “milieu” may offer an opportunity for determining the function of HDL. For example, HDL-C corrected by the SAA concentration correlates with risk better than HDL-C alone in several independent cohorts [77].

**Fig. 4:**
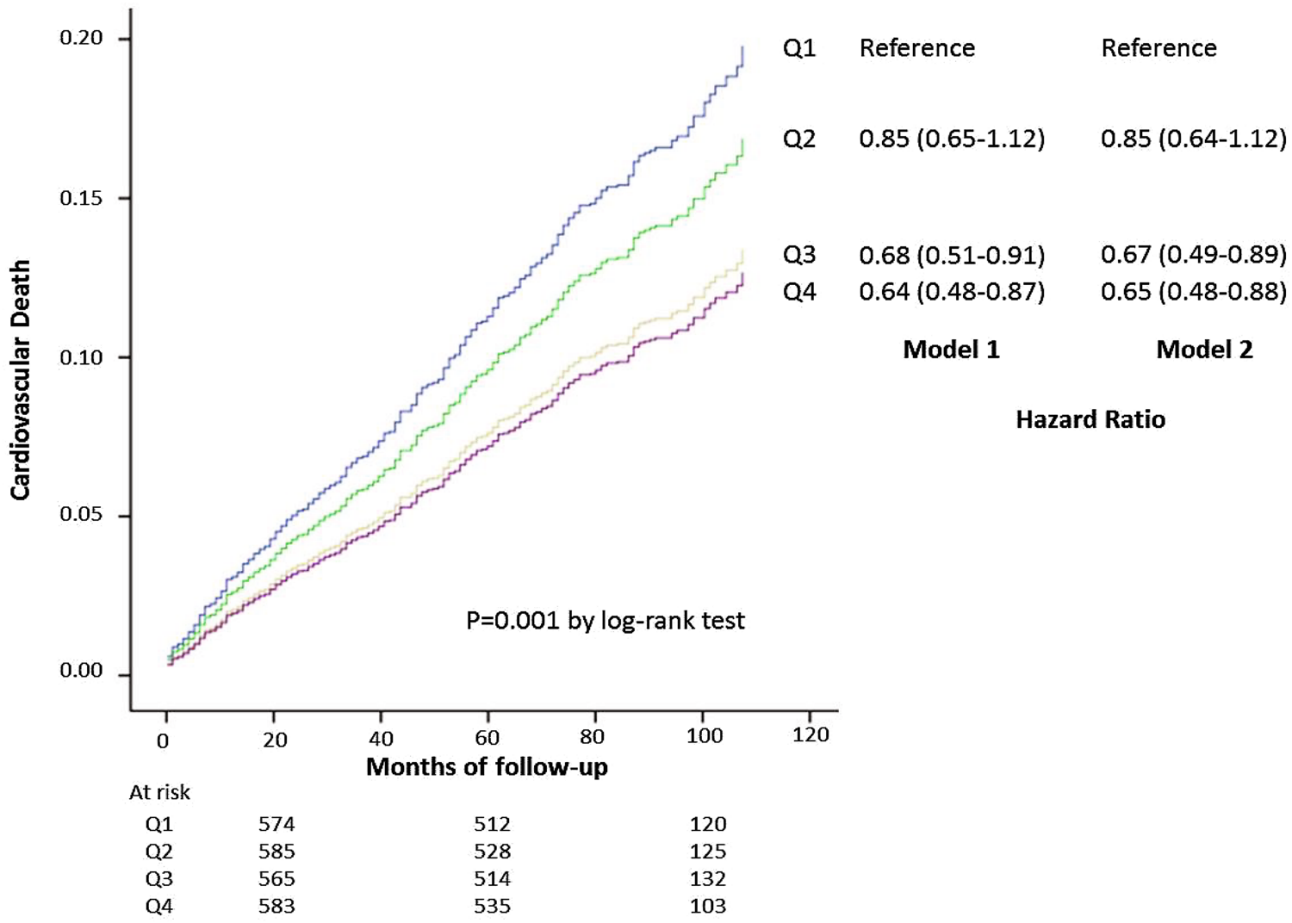
Cellular cholesterol efflux and cardiovascular mortality. Kaplan–Meier curves and hazard ratios by quartiles of cholesterol efflux capacity. Model 1: adjusted for age and gender; Model 2: also adjusted for use of lipid-lowering drugs, CHD (none, stable, unstable CHD, NSTEMI, STEMI), body mass index, hypertension, smoking, LDL-C/HDL-C ratio, triglycerides, metabolic syndrome/type 2 diabetes mellitus [26]. With permission of Springer [7]

Another way to determine the functionality of HDL in the laboratory, although one that requires more efforts, is measuring cellular cholesterol efflux. In this method, cultivated cells are enriched with labelled cholesterol and incubated with Apo B-free serum from the patient, and the rate of transfer of labelled cholesterol into the patient serum is determined. The rate of cholesterol efflux has been associated with the prevalence of CHD, independent of the HDL-C concentration [24], with cardiovascular events and deaths (Fig. 4) [25, 26]. In another study, cellular efflux was inversely correlated with prevalent atherosclerosis, but not with the long-term risk of cardiovascular events [78].

*In summary* The biological function of HDL is in part dependent on their protein composition. The properties of HDL are poorly reflected by their cholesterol content. In disease states, HDL may turn dysfunctional and can even have negative vascular effects.

## Should HDL-C be increased by therapeutic measures?

The positive vascular effects of HDL have been an attractive target for the treatment of chronic or acute vascular disease. Approaches to increase HDL include lifestyle modifications, conventional pharmacological therapy and novel therapeutic modalities which are still under scrutiny.

### Lifestyle

HDL-C can be increased through a number of lifestyle changes [79] (Table 2). The most important among these are increased physical activity and dietary changes. Lifestyle changes that increase HDL-C are also associated with reduced risk of cardiovascular disease [80]. The actual contributions made by increases in HDL-C are however not clear, because these interventions do not increase HDL-C in isolation, but influence metabolism in a variety of ways. Physical activity not only leads to an increase in HDL-C, but also to changes in HDL functionality [69]. At the same time, the metabolism of triglyceride-rich lipoproteins (fasting and postprandial) is improved [81, 82]. Yet, a number of other pathologies are also subject to positive changes (e.g. insulin sensitivity). We therefore conclude that the increase in HDL-C observed with lifestyle changes represents a marker of reduced cardiovascular risk.

**Table 2:** Influence of lifestyle modifications on HDL-C

Intervention	HDL-C increasein %	Effect
Physical activity	5–10	Increased LPL, pre-β-HDL, reverse cholesterol transport, protective lipoproteins
Smoking cessation	5–10	Increased LCAT, reverse cholesterol transport, inhibits CETP
Weight loss	5–20	Increased LCAT, LPL and reverse cholesterol transport
Alcohol consumption	5–15	Increased ABCA1, apo-A-I and paraoxonase, reduced CETP
Mediterranean diet (unsaturated fatty acids)	0–5	Increase in atheroprotective lipoproteins

*ABCA1* ATP-binding cassette transporter A1, *ABCG1* ATP-binding cassette transporter G1, *CETP* cholesteryl ester transfer protein, *LCAT* lecithin–cholesterol acyltransferase, *LPL* lipoprotein lipase

### Statins

Statins competitively inhibit HMG-CoA reductase, the rate-limiting enzyme for cholesterol biosynthesis, and they reduce LDL-C. Statins also lower triglycerides and slightly raise HDL-C. It is unlikely that this increase in HDL-C makes a substantial contribution to the reduction of events mediated by statins [83–85].

### Fibrates

Fibrates are agonists of the nuclear receptor PPAR-α (peroxisome proliferator-activated receptor alpha). Fibrates stimulated the expression of Apo A1 and via LXR the expression of ABCA1. Fibrates also up-regulate SR B1, which mitigates their HDL-raising effect, but is synergistic in activating RCT. Fibrates also activate lipolytic enzymes, lipoprotein lipase (LPL) in particular, which leads to increased catabolism of triglyceride-rich lipoproteins and an indirect increase in HDL-C.

A meta-analysis of 18 prospective, randomised outcome studies concluded that fibrates reduce the rate of severe cardiovascular events by 10% and the progression of albuminuria by 14% [86]. It is unclear whether the clinical effects of fibrates are due primarily to the increase in HDL-C, the decrease in triglycerides or the slight reduction in LDL. For patients with diabetes mellitus and statin therapy, fibrates have not been shown to provide any further reduction of risk [87–89]. Retrospective analyses of fibrate trials raise the possibility that reductions in risk can be achieved in subgroups with atherogenic dyslipidemia (high triglycerides and low HDL-C) [88, 90, 91]. In Phase 3 studies with new fibrates, this hypothesis is being tested prospectively. If these studies were successful, the use of fibrates in patients with high triglycerides and/or low HDL-C would be warranted.

### Niacin

Niacin and derivatives reduce triglycerides, VLDL cholesterol, LDL-C and lipoprotein (a), and they increase HDL-C. Among other factors, the effects are based on the inhibition of hepatic diacylglycerol acyltransferase 2, a key enzyme in the synthesis of triglycerides. Meta-analyses of studies with niacin (mostly without simultaneous administration of statins) indicate a decrease in cardiovascular events [92–94]. A subsequent meta-analysis of 11 outcome studies, which were mainly conducted in secondary prevention and included the AIM-HIGH-study [92], indicated that niacin reduced cardiovascular events and adverse coronary endpoints by 34 and 25%, respectively [94].

A combination of niacin with laropiprant was tested on 25,673 patients in the HPS2-THRIVE study [95]. No reduction in vascular events was observed, but the rates of severe side effects such as bleeding, myopathy, infections and diabetes mellitus increased. Since then, niacin preparations are no longer available in Europe. Based on the AIM-HIGH, ACCORD and HPS2-THRIVE studies, the Federal Drug Administration has come to the conclusion that there is no sufficient evidence for the reduction of cardiovascular events through the use of fibrates and niacin [96, 97], especially in conjunction with statin therapy.

### CETP inhibition

Cholesteryl ester transfer protein (CETP) mediates the exchange of cholesterol and triglycerides between various lipoproteins and thus plays an important role in RCT (Fig. 2) [98, 99]. The concept of CETP inhibition is based on the following observations: rodents are naturally CETP deficient, display high HDL-C and are resistant to diet-induced atherosclerosis. Patients with genetically reduced or absent CETP activity have high HDL-C. Inhibition of CETP has a positive influence on the lipoprotein profile in humans and could therefore show atheroprotective effects.

However, animal experiments have yielded contradictory results. In rabbits, CETP inhibition led to a reduction in atherosclerosis. In mice, both atheroprotective and atherogenic effects have been reported. Studies in humans have also been contradictory. On the one hand, patients with genetically reduced CETP serum concentrations appear to have a slightly reduced risk of coronary heart disease [99–102]. On the other hand, many studies in which CETP was tested in the plasma showed an inverse correlation between CETP and cardiovascular risk [37, 103]. Drug-based inhibition of CETP in humans leads to a significant increase in HDL-C and a drop in LDL-C [104, 105]. However, three out of four prospective studies with CETP inhibitors had to be terminated due to increased mortality in the treatment group or due to futility (Table 3). (https://investor.lilly.com/releasedetail.cfm?ReleaseID=936130) [106, 107]. The results of the large HPS-3/TIMI55 study, including over 30,000 patients, are pending.

**Table 3:** Prospective clinical intervention studies with CETP inhibitors

CETP inhibitor	Study	Patients	Started	Result
Torcetrapib	ILLUMINATE	ca. 15,000	2004	Cut short in 2006 due to increased mortality in the treatment group
Dalcetrapib	Dal-OUTCOMES	ca. 15,000	2008	Cut short in 2012 due to lack of effect
Anacetrapib	REVEAL	ca. 30,000	2011	Results expected in early 2017
Evacetrapib	ACCELERATE	ca. 11,000	2012	Cut short in 2015 due to lack of effect

*CETP* cholesteryl ester transfer protein

One reason for the disappointing performance of CETP inhibitors thus far could be the fact that the transfer of cholesteryl esters from HDL to LDL, and thus a pivotal step in RCT, is being inhibited. CETP inhibition probably also extends the lifespan of dysfunctional HDL. This may not only eliminate the postulated atheroprotective effect but even be harmful if HDL gained adverse properties [9, 74, 108]. Future strategies may include the identification of patients who benefit from CETP inhibition. Another alternative approach would be the development of new inhibitors that reduce CETP production or modification of CETP function [109].

### Other HDL-boosting drugs in development

Recent approaches primarily aim to optimise the function of HDL in RCT [110, 111]. Although HDL likely influences atherogenesis in a variety of ways, the focus is on the HDL-dependent aspects of RCT (Table 4). For instance, some such approaches use artificial discoidal HDL which, in addition to phospholipids, contain Apo A1 (CSL112) isolated from plasma, recombinant Apo A1 (CER001) or variant Apo A1 (Apo A1 Milano). Others generate pre-beta HDL via delipidation of the patient’s own plasma [111]. Both in vitro and in vivo, these approaches can achieve a transient acceleration of RCT, sometimes without causing any significant change in HDL-C. One problem is at present, however, that there is still no simple biomarker available for HDL functionality. The further development of Apo A1 Milano has been stopped in 2016 due to lack of efficacy [112]. However, the search for other possible ways to intensify RCT remains the subject of ongoing research.

**Table 4:** HDL-modifying substances in clinical trials

Drug (Manufacturer)	Properties	Development status
RVX-208 (Resverlogix)	Substance stimulates APOA1 transcription	Phase IIb IVUS study neutral [113]
CER-001 (Cerenis)	HDL mimetic produced from recombinant APOA1 complexed with phospholipids	IVUS-Study neutral, additional studies ongoing [114]
CSL111 (CSL Behring)	HDL mimetic produced from human APOA1 reconstituted with phospholipids	Further development cancelled
CSL112 (CSL Behring)	HDL mimetic produced from human APOA1 reconstituted with phospholipids	Replaces CSL 111
Recombinant APOA1 Milano; ETC-216, now MDCO-216 (The Medicines Company)	Natural mutation variant of APOA1, associated with a low rate of cardiovascular disease	Development stopped late 2016 111
APP018 (Bruin Pharma, licenced to Novartis in 2005)	Oral APOA1 mimetic (peptide), also known as D-4F	Current development status unclear
Delipidated HDL	Low-lipid HDL, produced by selective delipidation of HDL; can be used by autologous reinfusion (aphaeresis)	Current development status unclear
ACP-501 (AlphaCore Pharma, recently taken over by MedImmune)	Recombinant, human LCAT	Tested in Phase I study

*APOAI* apolipoprotein 1, *CETP* cholesteryl ester transfer protein, *LCAT* lecithin–cholesterol acyltransferase. Modified as per B.A. Kingwell et al. [110]

*In summary* An increase in HDL-C through lifestyle changes has positive effects and is recommended. This is especially true for smoking cessation and physical activity. Outcome studies with fibrates and with niacin have been conducted to evaluate the potential of HDL-C as a therapeutic target. However, since these drugs also cause drops in triglycerides and (to a moderate extent) LDL-C, neither fibrates nor niacin can conclusively value HDL-C as a drug target. There is no evidence for a reduction in clinical events through niacin or fibrates in combination with statins. In the studies available to date, increasing HDL-C with CETP inhibitors has not led to a reduction in clinical events. We conclude that HDL-C is not a target for drug-based therapy at the present time.

## Practical consequences

HDL play a central role in RCT. Large epidemiological studies have found an inverse relationship between the concentration of HDL-C in plasma and CHD. Mendelian randomisation studies show no protective effects of a genetically high HDL-C concentration. Cholesterol transported with HDL neither has any protective function nor reflects the functionality of HDL. HDL particles as such, as well as specific proteins and lipids in HDL, contribute to the classical functions of HDL in RCT, or mediate their anti-oxidative and anti-inflammatory properties. HDL composition is modified in complex ways in acute and chronic diseases. In this sense, it is misleading to interpret HDL-C as being equivalent to HDL or “good cholesterol”. The determination of pathophysiologically relevant lipids or proteins associated with HDL may come to replace HDL-C in the future, but at present such assays are not yet available for routine clinical use [9, 115]. The following clinical consequences result from these new findings:

- Low HDL-C is often a clinical indicator of disturbed metabolism of triglyceride-rich lipoproteins (e.g. in diabetes mellitus) or a chronic inflammation.
- The prognosis with regard to cardiovascular disease cannot be determined via HDL-C; the ratio of HDL-C to LDL-C may be misleading if HDL-C is high and if comorbidities like CHD, diabetes mellitus or chronic kidney disease exist.
- Increasing HDL-C through lifestyle factors such as physical activity and smoking cessation is associated with vascular protective effects.
- At present, HDL-C is not a target for drug-based treatment. The primary goal of lipid therapy is currently the reduction of LDL cholesterol.

Clearly, the “HDL-Cholesterol story” is not over but the recent findings set the stage for future research on this complex lipopoprotein.
